# Pharmaceuticals imports in Tanzania: Overview of private sector market size, share, growth and projected trends to 2021

**DOI:** 10.1371/journal.pone.0220701

**Published:** 2019-08-12

**Authors:** Dickson Pius Wande, Raphael Zozimus Sangeda, Prosper Tibalinda, Innocent Kairuki Mutta, Sonia Mkumbwa, Adonis Bitegeko, Eliangiringa Kaale

**Affiliations:** 1 Department of Pharmaceutics & Pharmacy practice, School of Pharmacy, Muhimbili University of Health and Allied Sciences, Dar es Salaam, Tanzania; 2 Department of Pharmaceutical Microbiology, School of Pharmacy, Muhimbili University of Health and Allied Sciences, Dar es Salaam, Tanzania; 3 Pharmaceutical R&D Laboratory, School of Pharmacy, Muhimbili University of Health and Allied Sciences, Dar es Salaam, Tanzania; 4 The Hubert Kairuki Memorial University, Dar es Salaam, Tanzania; 5 Tanzania Food and Drugs Authority, Ministry of Health, Dar es Salaam, Tanzania; 6 Department of Medicinal Chemistry, School of Pharmacy, Muhimbili University of Health and Allied Sciences, Dar es Salaam, Tanzania; University of Manchester, UNITED KINGDOM

## Abstract

**Background:**

To assess the extent to which foreign pharmaceutical imports vary from year to year and identifying leading generic and branded formulations, key countries and key importers of pharmaceuticals in private sector supply chain.

**Methodology:**

A systematic analysis of data for pharmaceutical imports from the Ministry of Health.Data from 2013 to 2016 fiscal years and relevant documents were accessed from the Tanzania Food and Drugs Authority (TFDA). Data cleaning was carried out to remove duplicate entries and to exclude pharmaceutical imports for individual uses, promotion purpose, donations, raw material, medical devices, government institutions and veterinary products.

**Results:**

A total of 397 different suppliers imported pharmaceutical in Tanzania mainland from 2013 to 2016 fiscal years. In the 2013–2014 fiscal year, the private sector suppliers imported pharmaceutical worth 216 U.S million dollars. India ranked as the first country for exporting highest value of pharmaceutical into the country. It displays a 54% cumulative market share of total imports from 2013–2016, followed by Egypt (11.7%), Switzerland and the USA hold 4.1% of cumulative market share. By 2020–2021 fiscal years, we forecast for imported pharmaceuticals to reach a total value of 906 U.S million dollars for the private sector supply chain. All analysis in this study and the forecasted figures are limited to private sector pharmaceutical supply chain only and does not include data for government pharmaceutical supply chain.

**Conclusions:**

Our result shows that the vast majority of pharmaceutical imports in the private sector supply chain are dominated by imports from India. India is competing with other countries such as Egypt, Switzerland, USA and South Africa among the top importing countries. There was almost an equal distribution of pharmaceutical for both communicable and non-communicable diseases. Data presented shows a growing trend for the market segment for medicines required for the management of non-communicable diseases. Generally, the private sector pharmaceutical market is keeping on rising at a rapid pace. By the year 2021, the growth is forecasted to increase by 28% compared to the current market value. The projected growth rate could be good news for foreign pharmaceutical companies seeking new sources of growth in international pharmaceutical trading. It is also good news to the poor patients if the availability of drugs previously unavailable in the country is significantly increased.

## Introduction

### Background

The United Republic of Tanzania is a union between Tanganyika and Zanzibar, which was formed in April 1964. Occupying an area of approximately 945,100 sq km, it is the largest country in East Africa. The pharmaceutical supply chain in Tanzania mainland is administered by the private distributors and public distributor (Medical stores department (MSD), which is an autonomous body under the Ministry of Health, Community Development, Elderly and Children). The private sector is predominant in urban and cities areas whereas the MSD covers throughout the country including urban, rural and hard to reach areas.

During the year the 1990s to 2005, domestic pharmaceutical production supplied approximately 30% of the domestic pharmaceutical market and about 10% of local production was exported[1]. Of recent, there has been a significant decline in domestic production of pharmaceuticals. In the year 2014, it was reported that domestic production accounts for only 12% of total demand[2], leading to raised importation, falling exports to less than US$1.7 million[3]and recording negligible average pharmaceutical export share of gross domestic product (GDP). Domestic manufacturers are mainly concerned with the production of generic medicines. The domestic pharmaceutical market is now supplied almost entirely by imports paid in US dollars[4]In 2018, there were more than twelve (12) registered pharmaceutical plants in Tanzania. Among the registered plants, only five were categorized as TFDA GMP compliant pharmaceutical plants. They are namely; Shelys Pharmaceuticals Ltd, Zenufa Laboratories Ltd, Pharma Centre, Prince Pharmaceuticals Ltd and Tanzania Pharmaceutical Industries Ltd (ARV production line)(5). Shelys Pharmaceuticals Ltd holds a 70% share of locally manufactured pharmaceutical in the country [5].

The dwindling trend for domestic production can be ascribed to lack of competitive advantage in the market price of locally produced pharmaceuticals compared to imported medicines[6] and difficulties in achieving economies of scale[7]. In addition, the cost of importing raw materials are becoming ever more expensive for local manufacturers as a result of the depreciating value of the local currency against the US dollar[8]. Conversely, even when pharmaceuticals are produced locally, there are sometimes doubts about quality given some reports of non-compliance with Good Manufacturing Practice (GMP)[9–11]. In a nutshell, the make or import dilemma[12], as faced by the other African countries, in Tanzania the importation of pharmaceuticals have become inevitable. The extent to which foreign pharmaceutical imports vary from different countries has not been studied in Tanzania. This study tries to generate a clear picture of the pharmaceutical market potential in private sector supply chain and identifying key pharmaceutical importers. This type of study can help to formulate strategies for improving pharmaceutical supply chains and forecast needs of the country or identifying potential areas of investment within the pharmaceutical sector. This could also help other foreign manufacturers to identify potential local technical representatives (LTR) for the pharmaceutical business opportunities in private sector pharmaceutical markets. Besides, the displayed data on market positions of different competitors could further help them to develop strategic business planning in the private sector pharmaceutical market in the country. Furthermore, from policymaking perspectives, the data presented could also benefit the Ministry of Health as well as the Ministry of Industry & Trade and Tanzania Investment Center. For the ministry of health, the data may be a useful reference for revising its national essential drug list and could be used for enticing industrialization in the country by the latter.

Thus, the aim of this paper is to shed light on the trend of pharmaceutical imports in Tanzania, with a special interest in private sector pharmaceutical supply chain. The specific goals are to 1) providing insights into new potential pharmaceutical markets; 2) identify key market players, trends and projections in the private sector pharmaceutical supply chain. The new insight on emerging trends could strengthen pharmaceutical business activities in the private market as well as may influence public policy-making regarding pharmaceuticals supply chain in Tanzania.

## Methods

A systematic analysis of pharmaceutical imports raw data from the Ministry of Health, Community Development, Elderly and Children were employed. Requests for raw data and relevant documents were submitted to the Tanzania Food and Drugs Authority (TFDA). The TFDA released selected attributes of the raw data for imported pharmaceuticals in the United Republic of Tanzania (mainland) from 2013 to 2016 fiscal years.

Data cleaning was carried out to remove duplicate data and to exclude pharmaceutical imports for individual uses, pharmaceuticals for promotion purpose, donations, raw material, medical devices, government institutions and veterinary products. Due to recent programmatic changes in the procurement policy for pharmaceuticals by the MSD, data presented here covers an exhaustive review for the pharmaceutical supply chain in the private health sector only. Data were analyzed using PivotTables (Microsoft office excel 2016). All forecasts were carried out using exponential smoothing method (13). The exponential smoothing model of forecasting was adopted on the basis that all-time series of numbers (years and corresponding import values) were available as per consistent and precise historical data on pharmaceutical importation accrued from TFDA.

## Results

A Systematic review of importation of various products that are currently under TFDA regulation from 2013–2016 is presented. The total import worth a cumulative value of 808 million USD was recorded from the analysed data. The incoterm adopted by TDFA for all international transactions is free on board (FOB). All data (values) are reported in USD currency. **Table 1** displays the values (USD) of various products that were imported into Tanzania mainland. Human medicinal products displayed the highest value compared to all other regulated products that were imported by the private sector pipeline.

**Table 1:** Categories of all products that were imported in the Private sector supply chain from 2013–2016.

Rank	Product	Frequency of importations	Sum of Total Amount (USD)
**1**	Human Medicinal Products	33636	**739,315,806.07**
**2**	Veterinary Pharmaceutical	2006	**65,701,743.94**
**3**	Promotion materials	14	**2,130,033.80**
**4**	Disinfectants	138	**433,684.76**
**5**	Herbal Drugs	71	**218,154.99**
**6**	Others	44	**151,200.47**
**7**	Vaccines	23	**52,246.42**
**8**	Raw Materials	465	**19,350.19**
**9**	Veterinary Vaccines	23	**965.00**
	**Grand Total (USD)**	**36421**	**808,023,185.65**

### Human medicinal products (HMPs)

Based on declared FOB values of commercial invoices by importers to Tanzania mainland, approximately 740 US million dollars were spent for the 2013–2016 fiscal years for importing HMPs in the private sector supply chain pipeline. **Table 2** summarises total value of pharmaceutical imports that were imported by private suppliers from 2013 to 2016 in Tanzania mainland.

**Table 2:** Total transactions and value in USD for fiscal years 2013–2016.

Fiscal Year	Frequency of Imports*	Sum of Total Amount (USD)	% Share
**2013–2014**	11098	216,269,806.98	29.3%
**2014–2015**	11549	229,246,160.66	31.0%
**2015–2016**	10989	293,799,838.43	39.7%
**Grand Total**	**33636**	**739,315,806.07**	**100.0%**

* Frequency: Number of importation documents that were submitted for TFDA approval for importation

#### Pharmaceutical imports value according to therapeutic category- Anatomical Therapeutic Classification (ATC) level 2 for the year 2013–2016

All pharmaceuticals imported under this period were classified up to level 2 of the ATC system of the WHO[13]. **Table 3** displays the top 20 ATC categories of the imported pharmaceutical for the 2013–2016 fiscal years. Antibacterial for systemic use was the leading ATC category with highest cumulative value (24.3%) followed by analgesics (17.8%), antimalarials (13.17) among the top three.

**Table 3:** Top 20 pharmaceutical products according to ATC category (Private sector supply chain pipeline).

Rank	ATC level 2	Sum of Total Amount (USD)	% Share
**1**	Antibacterial for systemic use	179,629,941.67	24.30%
**2**	Analgesics	131,569,045.62	17.80%
**3**	Antimalarials	97,378,220.21	13.17%
**4**	Antimycotics for dermatological uses	42,277,818.30	5.72%
**5**	Cough and cold preparations	29,685,354.44	4.02%
**6**	Anthelmintic	27,270,042.56	3.69%
**7**	Drugs for acid related disorders	21,760,639.42	2.94%
**8**	Antimycotics for systemic use	15,361,238.79	2.08%
**9**	Calcium channel blockers	14,851,010.57	2.01%
**10**	Drugs used in diabetes	14,823,456.99	2.01%
**11**	Antithrombotic agents	14,224,839.52	1.92%
**12**	Agents acting on the renin-angiotensin system	12,711,606.47	1.72%
**13**	Antivirals for systemic use	10,881,054.17	1.47%
**14**	Anti-asthmatics	8,644,265.96	1.17%
**15**	Anesthetics	8,181,669.03	1.11%
**16**	Multivitamins, combination	7,953,668.72	1.08%
**17**	Mineral supplements	7,687,668.47	1.04%
**18**	Cardiac therapy	6,403,215.40	0.87%
**19**	Corticosteroids for topical use	6,397,225.75	0.87%
**20**	Anti-inflammatory and antirheumatic products	6,084,449.92	0.82%
	Total	663,776,431.98	89.78%
	Other ATC categories	75,539,374.09	10.22%
	**Grand Total**	**739,315,806.07**	**100.00%**

### Pharmaceutical imports value and market share according to country of origin

During the period from 2013 to 2016 fiscal years, the private suppliers imported pharmaceuticals from 74 countries worldwide. **Table 4** shows the top 20 countries according to the declared FOB values in USD and cumulative market share of the total import value from 2013 to 2016. India, Egypt, Switzerland, USA and South Africa are ranked among the top 5 countries that had exported pharmaceutical with cumulative highest FOB values.

**Table 4:** Total pharmaceutical imports values and corresponding market share.

Rank	Country	Fiscal Years				
		2013–2014	2014–2015	2015–2016	Sum of Total (USD)	% Share
**1**	India	142,058,178.32	121,121,450.25	136,702,568.19	399,882,196.76	54.1%
**2**	Egypt	2,203,461.94	3,609,938.63	80,918,778.79	86,732,179.36	11.7%
**3**	Switzerland	5,162,127.23	19,577,206.29	5,745,538.10	30,484,871.63	4.1%
**4**	USA	11,166,938.10	17,946,448.26	1,089,339.95	30,202,726.31	4.1%
**5**	South Africa	12,601,181.90	10,897,627.60	5,842,507.91	29,341,317.40	4.0%
**6**	Malaysia	4,306,882.13	13,143,544.78	3,024,992.85	20,475,419.75	2.8%
**7**	Germany	2,198,505.62	3,769,803.23	11,590,016.45	17,558,325.29	2.4%
**8**	France	1,580,101.41	1,173,694.80	13,337,621.62	16,091,417.82	2.2%
**9**	Cyprus	6,372,627.71	6,139,387.66	3,506,521.59	16,018,536.95	2.2%
**10**	Bangladesh	12,457,538.15	1,275,153.42	749,324.78	14,482,016.34	2.0%
**11**	UK	2,157,515.80	6,326,728.48	2,146,403.70	10,630,647.98	1.4%
**12**	Kenya	826,521.18	1,150,039.16	7,274,382.94	9,250,943.28	1.3%
**13**	Pakistan	3,862,710.58	2,783,083.55	1,576,108.94	8,221,903.07	1.1%
**14**	South Korea	1,873,080.24	660,147.69	3,480,634.99	6,013,862.92	0.8%
**15**	Italy	628,101.38	2,189,122.73	2,874,851.37	5,692,075.48	0.8%
**16**	Netherlands	68,180.40	3,809,099.21	1,761,070.02	5,638,349.62	0.8%
**17**	China	965,734.53	1,885,881.28	2,194,252.61	5,045,868.42	0.7%
**18**	UAE	757,007.89	1,118,658.31	2,477,821.35	4,353,487.54	0.6%
**19**	Uganda	243,318.27	507,553.52	3,351,729.85	4,102,601.63	0.6%
**20**	Sweden	604,265.51	2,525,770.15	0.00	3,130,035.66	0.4%
	Total	212,093,978.27	221,610,338.99	289,644,465.98	723,348,783.25	97.8%
	Other countries	4,175,828.71	7,635,821.67	4,155,372.45	15,967,022.82	2.2%
	**Grand Total**	**216,269,806.98**	**229,246,160.66**	**293,799,838.43**	**739,315,806.07**	**100.0%**

*USA = United States of America*, *UAE = United Arab Emirates*, *UK =* United Kingdom

### Pharmaceutical frequency of imports according to the country of origin and sourced manufacturers

From 2013 to 2016 fiscal years, pharmaceuticals were imported from 74 various countries worldwide. **Table 5** shows the top 20 countries according to the counts or frequency of importation from a particular country. India (9810 counts) was leading followed by Kenya (9629 counts), Switzerland (1033 counts), South Africa (838 counts) and Cyprus (778 counts) among the top 5 countries that have a high frequency of exportation into Tanzania mainland.

**Table 5:** Total pharmaceutical import frequency from 2013–2016.

	Country	Frequency of importations (Counts)			
Rank		2013–2014	2014–2015	2015–2016	Grand Total
1	India	3209	3591	3010	9810
2	Kenya	3144	3018	3467	9629
3	Switzerland	325	406	302	1033
4	South Africa	297	291	250	838
5	Cyprus	261	260	257	778
6	Germany	223	329	224	776
7	UK	196	222	116	534
8	France	158	136	167	461
9	Egypt	126	125	104	355
10	China	113	118	113	344
11	UAE	40	71	176	287
12	USA	92	131	54	277
13	Netherlands	19	188	65	272
14	Pakistan	88	112	64	264
15	Malaysia	99	88	34	221
16	Bangladesh	99	80	40	219
17	Uganda	37	55	79	171
18	Sweden	61	93		154
19	Italy	37	49	30	116
20	Thailand	23	38	49	110

*USA = United States of America*, *UAE = United Arab Emirates*, *UK =* United Kingdom

**Table 6** displays the top 20 sourced manufacturers. Among the manufacturers that were sourced for importing pharmaceuticals, Cipla Limited (India) (11.17%) displayed the highest market share followed by Egyptian International Pharmaceutical Industries Company (10.77%), Astra lifecare (India) Private Limited (7.52%), Lincoln Pharmaceuticals Limited (India) (3.32%) and Hoe Pharmaceuticals SdnBhd (Malaysia) (2.24%) among the top 5 sourced Manufacturers.

**Table 6:** Top 20 sourced manufacturers.

Rank	Manufacturer	Grand Total	% Share
**1**	Cipla Limited	82,618,290.33	11.17%
**2**	Egyptian International Pharmaceutical Industries Company	79,610,209.14	10.77%
**3**	Astra lifecare (India) Private Limited	55,598,502.96	7.52%
**4**	Lincoln Pharmaceuticals Limited	24,518,093.70	3.32%
**5**	Hoe Pharmaceuticals SdnBhd	16,560,990.11	2.24%
**6**	Remedica Limited	12,375,472.23	1.67%
**7**	Denk Pharma GmbH & Co. KG	11,774,982.22	1.59%
**8**	Unichem Laboratories Limited	11,494,509.64	1.55%
**9**	Aventis Pharma Specialties	11,194,705.62	1.51%
**10**	Kopran Limited	10,684,986.38	1.45%
**11**	Fourrts (India) Laboratories Private Limited	10,482,416.41	1.42%
**12**	Sun Pharmaceutical Industries Limited	10,382,571.30	1.40%
**13**	Johnson & Johnson (PTY) Limited	9,692,203.00	1.31%
**14**	Warner-Lambert S.A Proprietary Limited	9,340,789.77	1.26%
**15**	Ajanta Pharma Limited	8,347,448.82	1.13%
**16**	Square Formulations Ltd	6,869,968.82	0.93%
**17**	Blue Cross Laboratories Limited.	6,513,902.85	0.88%
**18**	IPCA Laboratories Limited	6,227,287.74	0.84%
**19**	Msn Laboratories Private Limited	5,723,199.41	0.77%
**20**	Glenmark Pharmaceuticals Limited	5,173,636.81	0.70%
	Total	395,184,167.26	53.45%
	Others	344,131,638.80	46.55%
	**Grand Total**	**739,315,806.07**	**100.00%**

### Pharmaceutical imports value according to the dosage form

**Fig 1:**
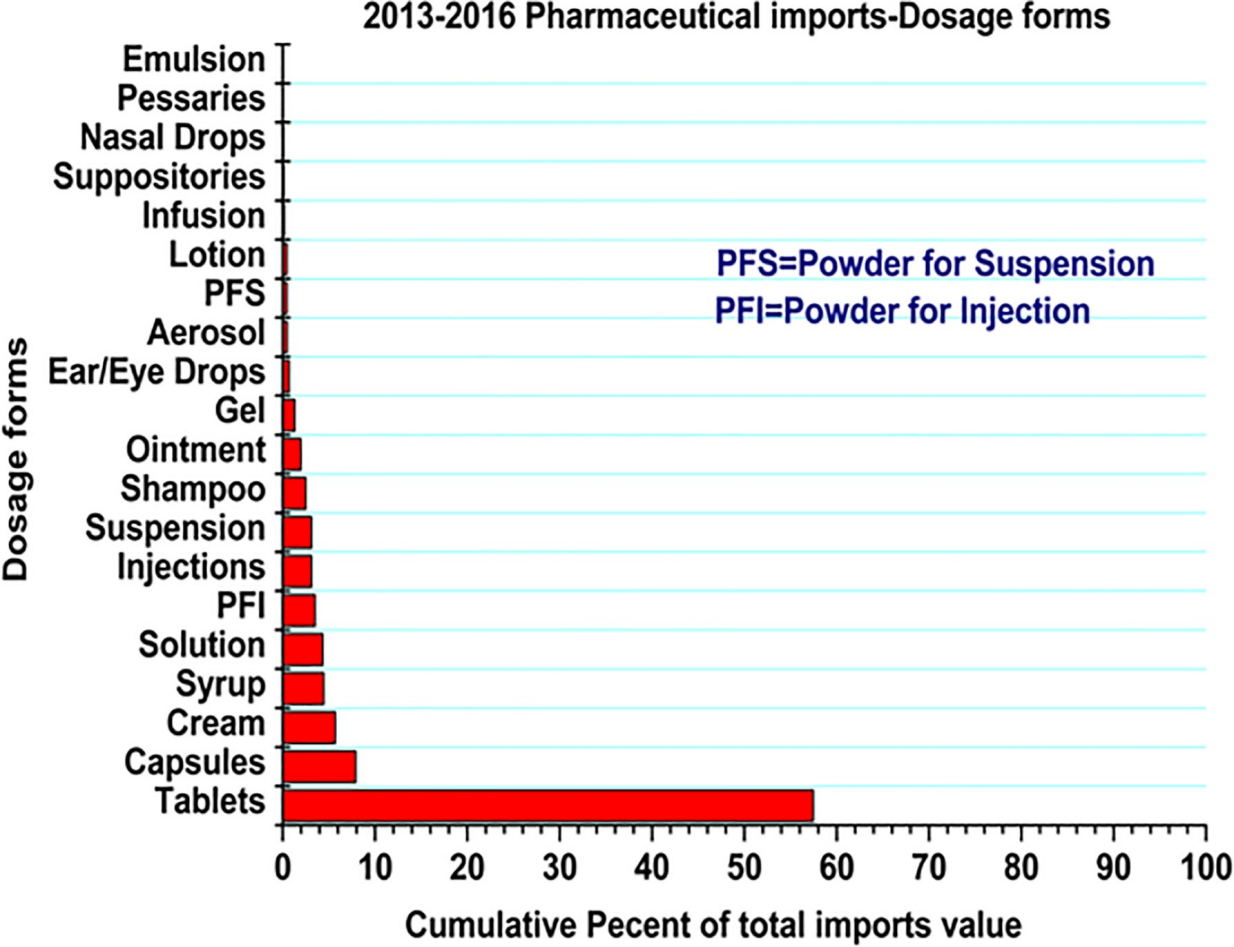
Pharmaceutical importations dosage-wise. Tablets were among the most imported dosage form with highest cumulative market share value (57.5%), followed by capsules (7.92%), Cream (5.68%), syrup (4.45%), powder for injection (4.35%) and injections (3.51%).

A total of 35 different dosage forms were imported into the country from 2013–2016 fiscal years. **Fig 1** summarizes the top 20 dosage forms that were imported into the country for this period.

### Pharmaceuticals importers—List of key local representatives/ distributors

**Fig 2:**
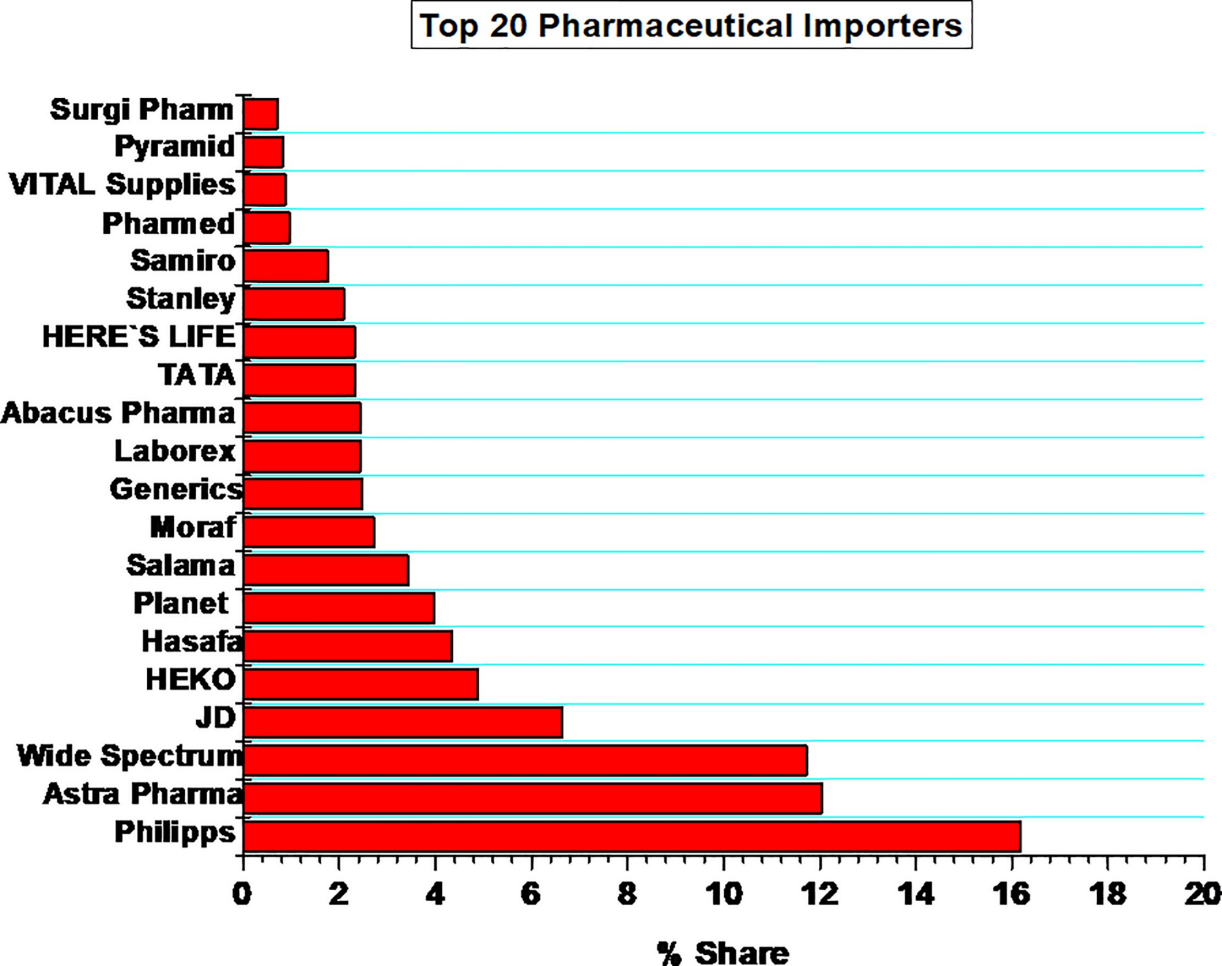
Top 20 local representatives/ importers in Tanzania mainland.

A total of 397 different company imported pharmaceutical in Tanzania mainland from 2013 to 2016 fiscal years. **Fig 2** displays the top 20 local technical representatives (LTR) in Tanzania mainland. Phillips Pharmaceutical (T) Limited had enjoyed the greatest market share (16.2%) followed by Astra Pharma (T) Limited (12%) and Wide spectrum (T) Ltd (11.7%), JD Pharmacy (6.9%) and HEKO Pharmaceuticals (5.2%) among the top five importers.

### Pharmaceuticals market trends

We analysed the cumulative market share for both brands (trade) name finished pharmaceutical formulations (BFPF) and generic finished pharmaceutical formulations (GFPF) that were imported into the country from 2013–2016 fiscal years. A total of 4286 BFPF and 928 GFPF were imported from different countries from 2013 to 2016 fiscal years. **Table 7 and Table 8** display top 20 BFPF and GFPF respectively, with the highest import value in U.S dollars.

**Table 7:** Pharmaceutical import value for BFPF from 2013–2016.

Rank	Brand	Manufacturer	Country	Total Amount (USD)	% Share
**1**	Lumartem	Cipla Ltd	India	78,238,328.84	10.58%
**2**	Epirax	EPICO	Egypt	77,618,440.00	10.50%
**3**	Asmol	Astra lifecare (India) Private Limited	India	51,055,289.83	6.91%
**4**	Kenazole	Brinton Pharmaceuticals Ltd.	India	16,075,116.52	2.17%
**5**	Dezor	Hoe Pharmaceuticals	Malaysia	15,738,080.80	2.13%
**6**	Zentel	GlaxoSmithkline (Kenya) Ltd	Kenya	15,576,275.82	2.11%
**7**	M-Cam	Unichem Laboratories Ltd	India	14,020,713.66	1.90%
**8**	Flucomol	Lincoln Pharmaceuticals Limited	India	11,945,992.40	1.62%
**9**	Clexane	Sanofi Winthrop Industrie	France	11,315,586.82	1.53%
**10**	NOSMOK	Unichem Laboratories Ltd	India	11,148,101.50	1.51%
**11**	Finazol	Fourrts (India) Laboratories Private Limited	India	10,246,701.62	1.39%
**12**	Benylin	Johnson & Johnson (PTY) Limited	South Africa	9,753,258.25	1.32%
**13**	Lokit	Kopran Limited	India	9,541,203.54	1.29%
**14**	Benylin 4 Flu	Johnson & Johnson (PTY) Limited	South Africa	9,509,816.61	1.29%
**15**	Metswift	Ind-Swift Limited	India	9,188,638.92	1.24%
**16**	Flexi	Square Formulations Ltd	India	9,054,304.38	1.22%
**17**	Coartem	Norvatis (India) Ltd	India	7,683,652.14	1.04%
**18**	Ketoral	BilimPharmaceticalsA.S	Turkey	7,155,364.16	0.97%
**19**	Meftal	Blue Cross Laboratories Limited	India	6,283,728.28	0.85%
**20**	Womiban	Blue Cross Laboratories Limited	India	5,037,529.89	0.68%
	*Total*			386,186,123.98	52.24%
	*Other Brands*			353,129,682.09	47.8%
	**Grand Total**			**739,315,806.07**	**100.0%**

EPICO = Egyptian International Pharmaceutical Industries Company

**Table 8:** Pharmaceutical import value for GFPF from 2013–2016.

Rank	Generic Medicine	Sum Total Amount	% Share
**1**	Artemether + Lumefantrine	89,840,781.40	12.15%
**2**	Paracetamol	79,124,851.94	10.70%
**3**	Chlordiazepoxide + Clinidium	77,618,440.00	10.50%
**4**	Ketoconazole	33,328,831.86	4.51%
**5**	Albendazole	26,327,246.13	3.56%
**6**	Metronidazole	26,009,350.47	3.52%
**7**	Diphenhydramine	23,782,107.25	3.22%
**8**	Chloramphenicol	23,694,352.13	3.21%
**9**	Miconazole	17,380,083.43	2.35%
**10**	Meloxicam	15,537,828.59	2.10%
**11**	Amoxicillin	14,117,702.19	1.91%
**12**	Amoxicillin and enzyme inhibitor	12,624,374.13	1.71%
**13**	Amlodipine	11,808,450.16	1.60%
**14**	Enoxaparin	11,385,473.24	1.54%
**15**	Omeprazole	11,137,234.04	1.51%
**16**	Metformin	10,249,127.08	1.39%
**17**	Aceclofenac	9,836,274.58	1.33%
**18**	Acyclovir	7,259,622.42	0.98%
**19**	Ketamine	7,191,101.50	0.97%
**20**	Mefenamic Acid	6,437,027.08	0.87%
	Total	514,690,259.60	69.6%
	Other Generics	224,625,546.47	30.4%
	**Grand total**	**739,315,806.07**	**100.0%**

The leading imported brands were Lumartem (Cipla-India) (10.58%), Epirax tablet (Eipico-Egypt)(10.50%) and Asmol (6.91) among the top three BFPF.The top 20 BFPF accounted for the total market share worth 386U.S million dollars of all imported pharmaceuticals for 2013-2016.The leading generic formulation among the top 20 GFPF with highest total import value is a fixed combination of Artemether and Lumefantrine (12.15%) followed by Paracetamol (10.7%), Chlordiazepoxide + Clidinium (10.5%) (a generic version of Epirax in Table 7), Ketoconazole (4.51%) and Albendazole (3.56%) among the top 5 imported GFPF. The top 20 GFPF accounted for cumulative market share worth 515U.S million dollars of all imported pharmaceuticals for 2013–2016 (Vs 740 U.S million dollars of total cumulative pharmaceutical import value). 65% off all top 20 BFPF were manufactured in India.

### Pharmaceuticals imports forecast

**Fig 3:**
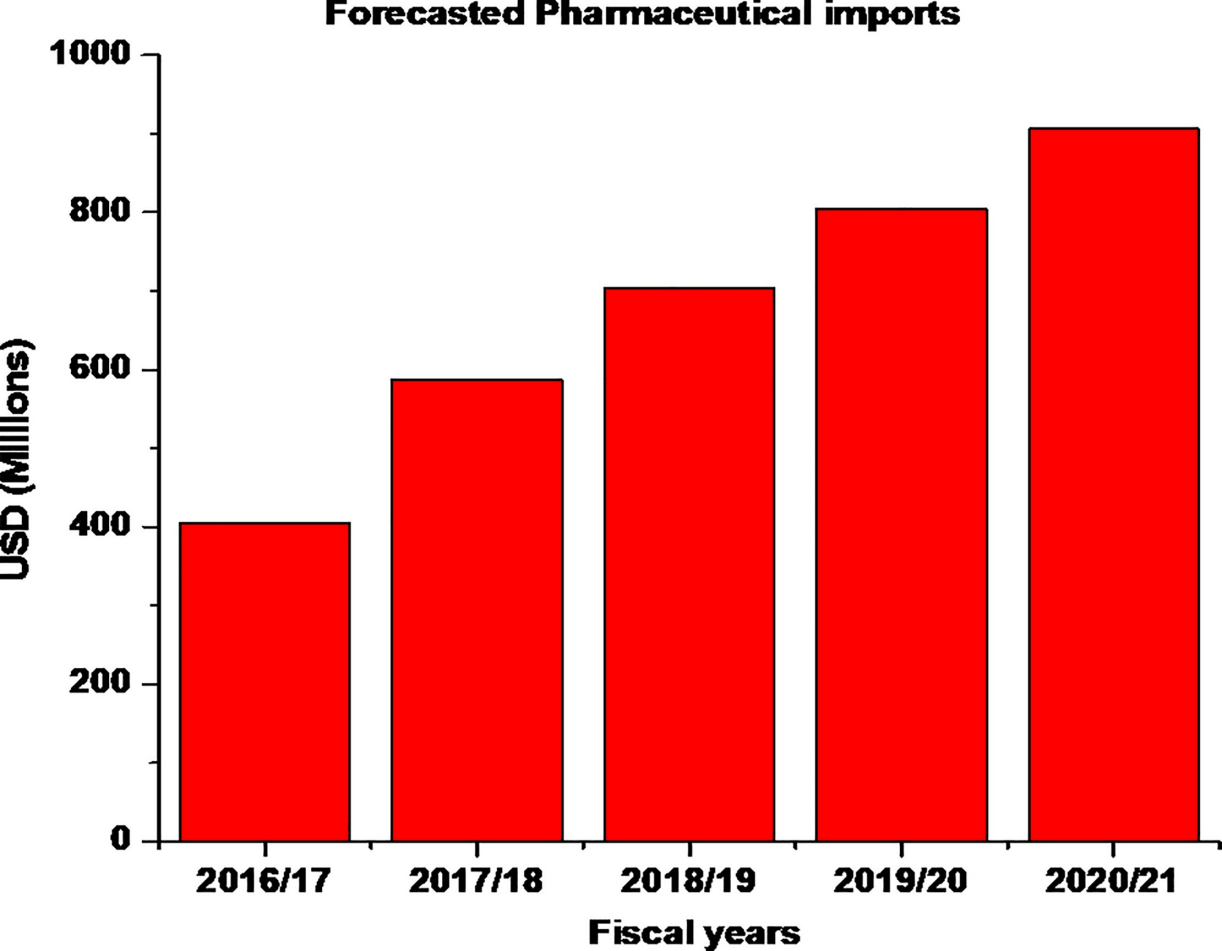
Pharmaceutical import forecast up to the year -2021(in Million USD).

We employed exponential smoothing[14], for forecasting pharmaceutical importation in the private sector supply chain. To remove the effect of inflation (deflation), the real pharmaceutical import figures were calculated by removing the pharmaceutical annual average consumer price index (CPI)[15]. The nominal pharmaceutical import forecast was then generated by returning the CPI numbers to real pharmaceutical import figures. **Fig 3** displays the forecasted import value for the fiscal years ending 2021 for the private sector supply chain pipeline in Tanzania mainland.

During the 2013–2014 fiscal years, pharmaceutical imports in Tanzania mainland reached 216 U.S million dollars. We forecast for the nominal pharmaceuticals imports to reach a total value of 704 U.S million dollars for all pharmaceutical import from private sector suppliers for the 2018–2019 fiscal year. By 2020–2021 fiscal years, it is projected that the pharmaceutical imports in the private sector supply chain will reach a value of 906 U.S million dollars. One of the limitations on these forecasted values is, for example, when the market drivers (e.g. improved local production capacity) change unexpectedly the projected values might be less accurate.

## Discussion

Among TFDA’s registered products that were cumulatively imported into Tanzania mainland for the period between 2013–2016 fiscal years, human medicinal products constitute a significant sizable market (740 U.S million dollars) compared with other TFDA registered products that were imported into the country. Veterinary pharmaceutical recorded the second largest value (65 U.S million dollars). The raw material for pharmaceutical manufacturing recorded a significantly lower value (19,350 U.S dollars). The value for importation has increased from 216 U.S million dollars in 2013 to 294 U.S million dollars in 2016 (Table 2), indicating approximately 17% annual growth rate of the total value of pharmaceuticals imports.

Based on level 2 of the ATC system, antibacterial for systemic use emerged as the leading ATC in terms of value of the total pharmaceutical imports (worth 180U.S million dollars) followed by analgesics (worth 132 U.S million dollars), antimalarials (worth 97 U.S million dollars) antimycotics for dermatological use (42 U.S million dollars) and Cough and cold preparations (30 U.S million dollars). Among the top 20 ATC categories, there is almost an equal distribution for both communicable and non-communicable diseases (9 ATCs falling under communicable versus 11 for non-communicable diseases- Table 3). There could be several factors that can be ascribed to this observation. It might be either an indication of an alarming rate for increasing burden of non-communicable disease in the country or there have been improved diagnostic techniques, changing prescription habits or improved pharmaceuticals supply chain in private sector pipeline.

Calcium channel blockers and drug used in diabetics ranked 9^th^ and 10^th^ respectively. Antibacterials and antimalarials are highly consumed in Tanzania because of a high number of infective diseases especially respiratory diseases and malaria [16,17]. From pharmaceutical markets perspectives, antibacterial were leading in the market, plain amoxicillin holds 1.9% market share. Amoxicillin in fixed combination with other antibacterials displayed 1.7% market share. Amongst antimalarials, Artemether in fixed combination with Lumefantrine holds 12.15% market share (Table 8).

The observed high values of imported calcium channel blockers and drug used in diabetics are associated with an alarming rate of heart-related diseases and diabetes as a result of sedentary lifestyles especially in urban and major cities in sub-Sahara countries[18–20]. This data is consistent with a published study which showed that obesity is on the rise in African countries including Tanzania, it was revealed that from the year 1992 to 2005 the prevalence of overweight/obesity increased by nearly 35.5% and the prevalence of obesity was as high as 32% in urban Tanzania, compared with 12% in rural Tanzania[21].

Obesity is a major risk factor for the development of various non-communicable diseases, such as heart disease and diabetes. The number of diabetes patients in Tanzania is forecast to increase to 3.8million by 2035 from 1.7million in 2014[22]. From pharmaceutical markets perspectives, this represents a growing market for drugs used in diabetics, currently, among drugs used in diabetes, plain metformin is leading with 1.39% market share (**Table 8**)).

Analgesics were the second ATC with the highest market share in the country (Table 3). There are two possible reasons, firstly is due to their affordable pricing they easily associable for self-medication, secondly, they have well-developed distribution networks through pharmacies, ADDO, traditional drug shops (popularly known as ‘*dukala dawabaridi’*) and in supermarkets and local shops. Although this represents a good opportunity for pharmaceutical markets in the country, from a public health perspective this translates into a major risk for kidney diseases. Long term consumption of analgesics especially for self-medication, is a major risk factor for the development of chronic kidney disease (CKD)[22–24]. A study published in the year 2014 showed that Tanzania is at a higher risk for explosive growth in the burden of CKD[25].

Antimycotics for both dermatological and systemic use also represent a significant proportion in pharmaceutical markets in the country with 5.72% and 2.08% market share respectively (Table 3). The increased use of antimycotics infections has been linked to increasing use of the broad-spectrum antibiotics, anticancer therapy and increase in prevalence of immunocompromised infections such as acquired immune deficiency syndrome (AIDS)[26,27]. In 2016, it was estimated that 1.3 million peoples were living with HIV with HIV prevalence of 4.7% in the country[28]. The fact that systemic fungal infection remains one of the major opportunistic infection for people living with HIV, the market segment for antimycotics is expected to grow significantly.

Cough and cold preparations represent another ATC category with a significant contribution to the pharmaceutical markets in the country. The growing trend for this segment could be due to well-developed distribution networks similar to that of analgesics. In addition, the fact that lower respiratory infections were ranked as the second leading cause of all dearth in the country[17], we expect the pharmaceutical market for this segment to grow steadily. Among the drugs for the respiratory system; salbutamol was the leading molecule Asmol, **Table 7** and Diphenhydramine in **Table 8**

The antimalaria drug Artemether and Lumefantrine (ALu) in fixed-dose combination has become the leading in both BFPF (Lumartem, **Table 7**) and GFPF. The impetus for massive importation of ALu could be driven by the fact that in the current malaria treatment guidelines, ALu is the first line drug of choice for management of uncomplicated malaria[29], secondly, the support from Global Fund for subsidised ALu in both private and government facilities throughout the country[30–32] and/or mushrooming of private drug shops that have contributed to increased consumption of certain essential medicines in the country including antimalarials [33–36]. The subsidised ALu has made a substantial shift in malarial management from the time-tested old molecules (e.g. quinine and its derivative products). The current policy requires combination therapy for management of malaria; all other single molecules (e.g. Chloroquine, Amodiaquine e.tc) have been phased out. Only parenteral quinine has been reserved for the treatment of severe malaria[29].

Among the antidiarrhea drugs; while all the market segments were still growing, metronidazole market grew significantly whereas the fixed combination products like Norfloxacin and Tinidazole declined significantly in the year 2013–2016 (did not appear either in **Table 7** or **Table 8**).

Generally, when we compare the trend among prescription-only medicines and over the counter medicine (OTC) for the top 20 GFPF and BFPF, the prescription-only medicine displayed high import values in both categories. For the BFPF there were no OTC among the first top 10, only four OTCs appeared on the 12^th^(Benylin),14^th^(Benylin 4 Flu) 19^th^(Meftal) and 20^th^(Womiban). For the GFPF category, prescription-only medicines display high prevalence than the OTC with only six OTCs appearing on 2^nd^ (Paracetamol) 5^th^ (Albendazole), 7^th^ (Diphenhydramine) and 10^th^ (Meloxicam) among the first top 10. Therefore, the OTCs display high prevalence in GFPF over the BFPF. (**Table 7 and Table 8**)

Currently, there is no law or regulations that govern neither procurement nor the pricing of pharmaceutical in the private sector. The procurement act only governs government procurements. Secondly, although the standard treatment guidelines are in place, they are not enforced in the private sector. It’s not an uncommon in Tanzania to find that most people practising self-medication using prescription-only medicines[37]. This might have an influence on the observed pattern of private sector imports.

As it has continued to maintain a pre-eminent role as the pharmacy of the developing world[38], India ranked as the first country for exporting highest value of pharmaceutical into the country. It enjoyed a 54% total market share of total imports from 2013–2016, followed by Egypt (11.7%), Switzerland and the USA hold 4.1% of market share (**Table 4**). The top 20 countries hold 97.8% market share, while the rest 54 countries contributed to a 2.2% share of total import values from 2013–2016 fiscal years. On the other hand, in terms of frequency (‘counts’) of importation, India has emerged as the leading country, followed by Kenya and Switzerland as the second and third respectively (**Table 5**). Egypt was ranked the 2^nd^ in table 4 but it appears to decline in Table 5 in which it was ranked the 9^th^, this depicts that there was a relatively low frequency of importing relatively high value (in USD) of pharmaceuticals from Egypt. USA, Malaysia and Bangladesh displayed a similar trend. Kenya (which was ranked the 12^th^ in (**Table 4**) was ranked the 2^nd^ (**Table 5**). In this case, data shows that pharmaceuticals that were imported from Kenya worth low total value in U.S dollars. This further suggests that pharmaceuticals were frequently imported from Kenya than from other countriesprobably due to the given geographic proximity. UAE and Uganda displayed a semblable trend.

Among the top 20 importers, Phillips Pharmaceuticals (T) LTD displayed the highest market share, followed by Astra Pharma(T) LTD and Wide Spectrum. The majority of imports were supplied (sourced) from Cipla Limited, Egyptian International Pharma, Unichem Laboratories Limited and Johnson & Johnson (PTY) Limited (**Table 6**). All key distributors (importers) are based, and operate their business in Dar es Salaam, the economic centre and the largest city in Tanzania. Owing to the obvious advantages of tablets over other dosage forms[39], tablets constituted higher percentage of total import value. Capsules emerged as the second dosage form with high import value followed by creams, syrup, solutions and powder for injections. The inference from this data could be an eye opener to the would-be investors in pharmaceutical plants to invest/concentrate more in manufacturing these dosage forms. Furthermore, due to the fact that tablets and capsules are relatively cheaper in production and easily adaptable to GMP, local manufacturers should explore the economies of scale and increase the capacity to accommodate for the high demand of these dosage forms in the country.

Generally, in Tanzania pharmaceutical markets, generic imported pharmaceuticals have higher market share than branded imported pharmaceuticals, this is evident when we compare the contribution of top 20 categories in (**Table 7)** and (**Table 8)** against total pharmaceutical import values. The inference from this data is clear; GFPF predominate Tanzania’s pharmaceutical market as a result of low purchasing power by patients and/or lack of good marketing strategies (poor promotion) for branded BFPF manufactured by MNCs.

Recently, there has been a significant increase in budgetary funding for the government health care delivery[40,41]; this implies that the government might increase its share of pharmaceutical imports over the private sector, which might slightly lower demand in private sector due to improved accessibility and availability of pharmaceuticals in Government facilities. Therefore, our forecasted value for the private sector supply chain pipeline could be slightly lower than projected value, but the total actual national demand will not be affected, and in fact, the total combined value for pharmaceutical imports (i.e. from the private sector and government supply) will keep on increasing.

## Conclusion

Our result shows that the vast majority of pharmaceutical imports in the private sector supply chain are dominated by imports from India. India is competing with other countries such as Egypt, Switzerland, USA and South Africa among the top importing countries. There was almost an equal distribution of pharmaceutical for both communicable and non-communicable diseases. Data presented shows a growing trend for the market segment for medicines required for the management of non-communicable diseases.

Generally, the private sector pharmaceutical market is keeping on rising at a rapid pace. By the year 2021, the growth is forecasted to increase by 28% compared to the current market value. The growth could encompass more generic pharmaceuticals than branded pharmaceuticals; this is suggestive of the relatively high price of branded pharmaceuticals compared to generics. The projected growth rate could be good news for foreign pharmaceutical companies seeking new sources of growth in international pharmaceutical trading. It is also good news to the poor patients if availability of drugs previously unavailable in the country is significantly increased.

## Supporting information

S1 File (XLSX)
